# Ratios of CSF proteins reflect cognitive function in ALS

**DOI:** 10.1186/s13195-026-01976-y

**Published:** 2026-01-31

**Authors:** Linn Öijerstedt, Sára Mravinacová, Jennie Olofsson, Louisa Azizi, Sofia Bergström, Solmaz Yazdani, Nina De Vita, Inci S. Aksoylu, Juliette Foucher, Alexander Juto, Ulf Kläppe, Peter Nilsson, Anna Månberg, Caroline Ingre

**Affiliations:** 1 Department of Clinical Neuroscience, Karolinska Institutet, Stockholm, 17177 Sweden; 2 Department of Neurology, Karolinska University Hospital, Stockholm, 17176 Sweden; 3 Department of Protein Science, KTH Royal Institute of Technology, SciLifeLab, Stockholm, 17165 Sweden

**Keywords:** Amyotrophic lateral sclerosis, Cognitive impairment, ECAS, Proteomics, Ratios

## Abstract

**Background:**

Cognitive impairment is a recognised feature of neurodegenerative diseases, including amyotrophic lateral sclerosis (ALS). Despite advances in understanding cognitive impairment in ALS, no fluid biomarkers reliably predict these changes. Prior research in Alzheimer disease (AD) has demonstrated that CSF protein ratios enhance biomarker accuracy by mitigating inter-individual variability, improving diagnostic precision. In AD, ratios involving synaptic markers have shown stronger associations with cognitive outcomes than single proteins, motivating evaluation of a similar ratio-based approach in ALS.

**Methods:**

Building on findings from the AD field, we analysed 47 CSF proteins, suggested to be associated to neurodegeneration, in 66 patients with ALS and explored protein ratios to evaluate their utility in detecting cognitive impairment, hypothesising shared mechanisms between neurodegenerative diseases. Elastic net regression identified the most predictive protein pairs associated with cognitive impairment, assessed with the Edinburgh Cognitive and Behavioural ALS Screen (ECAS).

**Results:**

Elastic net identified seven single proteins (NEFM, NPTX2, GAP43, IGFBP4, IGFBP7, SPP1, CDH8) and eight protein pairs associated with ECAS total score. Ratios were generally more informative than individual proteins, with PTPRN2/GAP43 showing the strongest association with ECAS scores, indicating an enhanced ability to capture cognitive changes. Several of the proteins in the most predictive pairs have previously been implicated to associate to cognitive impairment in AD.

**Conclusion:**

Our findings indicate that protein ratios outperform single-protein analyses in detecting associations with cognitive impairment, aligning with advancements in AD research. By extending the concept of CSF protein ratios from AD to ALS, this study highlights shared pathological mechanisms and suggests that similar proteins are linked to cognitive dysfunction in both diseases.

**Supplementary Information:**

The online version contains supplementary material available at 10.1186/s13195-026-01976-y.

## Background

Cognitive dysfunction is a recognised feature of numerous neurodegenerative diseases, not only dementia disorders. In amyotrophic lateral sclerosis (ALS), cognitive impairment spans a spectrum from subtle executive dysfunction to full overlap with frontotemporal dementia (ALS-FTD) [1, 2]. Despite advances in understanding ALS-related cognitive impairment, efforts to translate this knowledge into clinical tools have lagged behind. In particular, biomarker research in cerebrospinal fluid (CSF) has increasingly focused on protein levels and their dynamics, with promising findings from studies of neurodegenerative diseases like Alzheimer disease (AD) [3, 4].

However, currently there are no fluid biomarkers that predict nor identify cognitive impairment in ALS. Filling this gap is critical as cognitive impairment in ALS has significant implications for disease management, affecting patients’ ability to make informed decisions regarding care, treatment, and participation in clinical trials. Biomarkers that detect early cognitive dysfunction could also enable more targeted and timely interventions, potentially improving quality of life. Moreover, cognitive impairment is linked to shorter survival in ALS [5], highlighting the prognostic value of identifying at-risk individuals early in their disease course. Together, these factors underscore the need for fluid biomarkers that not only detect neurodegeneration but also track its impact on cognitive function.

Emerging evidence suggests that interindividual variability in CSF protein levels can mask disease-related changes, complicating the interpretation of biomarker data [6]. By adjusting for this variability, protein ratios have been shown to enhance diagnostic and prognostic accuracy in AD [7, 8]. In addition to their diagnostic utility, CSF biomarkers can provide valuable insights into disease mechanisms. For example, alterations in proteins such as neurofilaments, which are consistently elevated in ALS, reflects axonal damage while proteins like chitinases may capture central nervous system inflammation relevant to ALS pathophysiology [9, 10]. By combining proteins associated with different disease mechanisms, a synergistic or additive effect could enhance their potential as a biomarker.

Building on the prior findings in AD, here we extend the concept of protein ratios to ALS, suggesting a shared utility of protein ratios as biomarkers for neurodegenerative cognitive dysfunction. We investigated a panel of CSF proteins in a well-characterized cohort of patients with ALS. Specifically, we focused on assessing the ability of these proteins to detect cognitive impairment, both individually and through protein ratios.

## Methods

### Cohort and data collection

Participants were recruited from the ALSrisc study, an ongoing longitudinal cohort study at Karolinska University Hospital in Stockholm, Sweden [11]. We included patients with a diagnosis of ALS, an available CSF sample and assessment of cognitive function within a year from sample collection. The following clinical and phenotypic data were collected: age at sampling, sex, site of onset and *C9orf72* repeat expansion. Disease progression was monitored using the ALS Functional Rating Scale–Revised (ALSFRS-R), a standardised instrument widely used to quantify functional decline in ALS [12]. Cognitive function was evaluated using the Edinburgh Cognitive and Behavioural ALS Screen (ECAS), a validated screening tool specifically developed for ALS populations [13]. ECAS administration takes approximately 15–20 min and assesses both ALS-specific (language, verbal fluency, executive function) and ALS-non-specific domains (memory, visuospatial function). The ECAS score collected closest to CSF sampling date was included. The median time between CSF sampling and ECAS was 7 weeks (range 0–35 weeks). No subject had ECAS assessed prior to sample collection. An ECAS total score below 108 will herein be referred to as “cognitive impairment” [14].

### Sample collection

CSF samples were collected between 2019 and 2021 via lumbar puncture into polypropylene tubes and processed immediately by centrifugation at 963 ×g for 10 min at room temperature. The resulting supernatant was aliquoted into cryotubes and stored at − 80 °C until analysis. Prior to protein analysis, the samples were stratified into 96-well PCR plates using a constrained randomisation approach based on age, and sex.

### Protein analysis

The analysis was restricted to a predefined panel of 47 CSF proteins. The proteins were selected based on prior in-house neuroproteomic studies, both published [15–22] and unpublished, identifying proteins and protein ratios with enhanced ability to reflect neurodegenerative pathology and related biological processes in CSF, where ratio-based readouts outperformed single-protein measures. The present study was designed as a hypothesis-driven evaluation of whether this ratio-based approach generalises to ALS, with particular emphasis on cognitive impairment. Corresponding polyclonal rabbit antibodies were sourced from the Human Protein Atlas (www.proteinatlas.org), except for progranulin (GRN) (AF3156-SP, R&D Systems) and apolipoprotein E4 (APOE4) (M067-3, MBL Life Science) antibodies (Supplementary Table 1). To create the bead array, each antibody was individually immobilised onto uniquely color-coded, carboxylated magnetic beads (MagPlex, Luminex Corp.) as previously described [23].

CSF was processed according to a previously described protocol [23]. In brief, the samples were diluted 1:2 and labelled with biotin (NHS-PEG4-biotin, A39259, ThermoFisher Scientific) followed by heat-treatment at 56 °C for 30 min. Incubation with beads was performed overnight at room temperature in 384-well plates and bound proteins detected using a streptavidin-conjugated fluorophore. Fluorescence signals, corresponding to median fluorescence intensity per bead ID and per sample, were acquired using a FlexMap 3D instrument (Luminex Corp.), providing relative quantification of protein levels.

### Statistical analysis

All data pre-processing, analysis and illustrations were performed in R Studio version 4.3.1. Protein level data was adjusted for delayed instrument readout using robust linear regression as described previously [3]. All protein levels were log2 transformed and scaled to zero mean and unit variance prior to statistical analysis. Assumptions for statistical models were assessed visually by residual plots (independence and equal variance) and normal probability plots (normality). The Wilcoxon rank-sum test was used to compare differences between sexes and the Kruskal–Wallis test was applied to assess differences across groups for site of symptom onset and genetic status.

#### Correlation and hierarchical clustering

Spearman’s rank correlation coefficients (*ρ*) were computed to assess the co-variation of CSF protein profiles. Hierarchical clustering was performed using Ward’s minimum variance method on a correlation-derived dissimilarity matrix defined as d = 1 − *ρ*.

#### Elastic net

Elastic net regression is a statistical modelling technique that addresses the challenges of analyzing datasets with a large number of interrelated variables [24]. It combines two forms of regularization, those used in ridge regression and Least Absolute Shrinkage and Selection Operator (LASSO), to improve model performance and interpretability. Ridge regression reduces overfitting by shrinking the size of all regression coefficients, but it does not eliminate any variables. In contrast, LASSO can shrink some coefficients to exactly zero, thus performing both shrinkage and variable selection, i.e. effectively selecting a smaller subset of variables that contribute most to the outcome. Elastic net introduces a mixing parameter, α (alpha), which controls the balance between ridge (α = 0) and LASSO (α = 1) penalties. By adjusting α, elastic net provides a flexible framework that combines the strengths of both methods. This makes elastic net especially effective when working with high-dimensional data and/or highly correlated predictors, as was the case in our study. In addition to the mixing parameter, elastic net al.so includes a second parameter, λ (lambda), which controls the overall strength of the penalty applied to the model. Larger values of λ impose stronger penalties, leading to greater shrinkage of the regression coefficients producing simpler, more constrained models (Supplementary Fig. 1).

##### Data setup

We partitioned the dataset into 70% for training and 30% for testing. The training data included 48 patients with a median ECAS total score of 109, the test set of 18 patients with a median ECAS total score of 108.5.

##### Tuning procedure

A grid search over α values (step size 0.01) ranging from 0 (Ridge) to 1 (LASSO) was conducted using cross-validation to identify the optimal mixing parameter. The best α was selected based on the minimum cross-validation error. Subsequently, with the optimal α, a ten-fold cross-validation was performed to select the optimal regularisation parameter λ (λ_min_​) in order to obtain the ideal trade-off between bias and variance. A cross-validation plot was used to support the tuning process (Supplementary Fig. 2).

##### Model fitting and evaluation

The elastic net model was fit on the training dataset using the optimal α and λ values. The root mean squared error (RMSE) for the training set was computed to assess model fit and potential overfitting. RMSE is a commonly used metric that quantifies the average magnitude of prediction error, with lower values indicating better model performance. Similarly, RMSE was calculated for the test set to evaluate prediction accuracy.

#### Regression models

Linear regression models were constructed to evaluate the association between candidate proteins and cognitive scores (ECAS total score). For each protein, a separate model was fitted, with ECAS total score as the outcome variable and protein levels as predictor with adjustments for age at sampling, sex, site of onset and ALSFRS-R total score. Model performance and generalisability were assessed using 10-fold cross-validation.

$$\begin{aligned} \mathrm{ECAS}_i\;=&\:\beta_{0}\;+\;\beta_{1}\;\cdot\;\mathrm{Protein}_{i}\;+\;\beta_{2}\;\cdot\;\mathrm{Age}_{i}\;+\;\beta_{3}\;\cdot\;\mathrm{Sex}_{i}\;\\&+\;\beta_{4}\;\cdot\;\mathrm{SiteOfOnset}_{i}\;+\;\beta_{5}\;\cdot\;\mathrm{ALSFRS}\;-\;\mathrm{R}_{i}\;+\;\epsilon_{i} \end{aligned}$$

To assess the ability for candidate proteins to predict cognitive status (cognitive impairment vs. no cognitive impairment), logistic regression models were constructed. All models were adjusted for age at sampling, sex, site of onset, and ALSFRS-R total score. Model performance was evaluated by receiver operating characteristic (ROC) curve and area under the curve (AUC).

$$\begin{aligned} \mathrm{log}\left(\frac{P\left(CI_{i}\right)}{1-P\left(CI_{i}\right)}\right)=&\:\beta_{0}\;+\;\beta_{1}\cdot\;\mathrm{Protein}_{i}\;+\;\beta_{2}\;\cdot\;\mathrm{Age}_{i}\;+\beta_{3}\;\cdot\;\mathrm{Sex}_{i}\;\\&+\;\beta_{4}\;\cdot\;\mathrm{SiteOfOnset}_{i}\;+\;\beta_{5}\;\cdot\;\mathrm{ALSFRS}\;-\mathrm{R}_{i} \end{aligned}$$

## Results

The final cohort included 66 patients (mean age = 66 ± 11.4 years, 39% female) of whom 44% had an ECAS total score below 108 (Table 1). The ECAS total score was not correlated to age or disease duration. The majority of patients had a spinal onset (68%), and nine individuals (14%) carried a *C9orf72* repeat expansion.

**Table 1:** Cohort. Characteristics of the patients included in the study. N, number; SD, standard deviation; *C9orf72*, C9 open reading frame 72; ECAS, Edinburgh cognitive and behavioural ALS screen; IQR, inter quartile range; ALSFRS-R, ALS functional rating scale–revised

Variable	*N* = 66
Age at sampling, mean years (SD)	66 (11.4)
Sex, *N* females (%)	26 (39)
Site of onset, *N* (%)	
Spinal	45 (68)
Bulbar	19 (29)
Other^a^	2 (3)
Disease duration^b^, median weeks (IQR)	49 (32)
*C9orf72* repeat expansion, *N* (%)	9 (14)
ECAS, median (IQR)	109 (21.5)
ECAS < 108, *N* (%)	29 (44)
ALSFRS-R, median (IQR)	41.5 (8.25)

^a^ respiratory

^b^ defined as time from symptom onset to sampling

### Neurofilament levels do not correlate to known markers of cognitive function

**Fig. 1:**
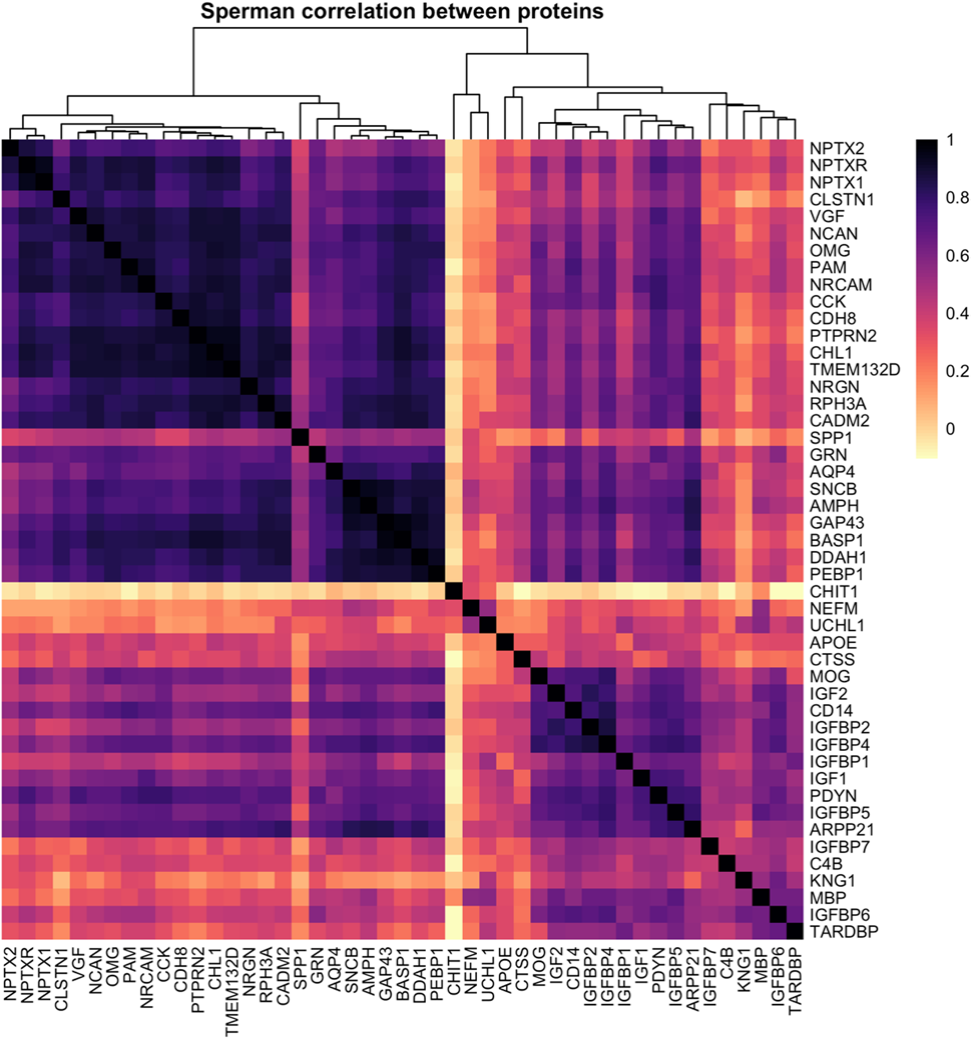
Correlation matrix. Spearman’s rank correlation and hierarchical clustering using the Ward’s minimum variance method. Heatmap colours (darker indicate stronger correlation) represent signed Spearman correlation coefficients. Clustering was performed using Ward.D2 on distances

Pairwise correlation analysis of the 47 CSF proteins revealed two main protein clusters, one with strongly correlating proteins (*n* = 26, median ρ 0.81, IQR 0.14) including proteins commonly regarded as markers for dementia and cognitive function such as neurogranin (NRGN), beta-synuclein (SNCB) and neuromodulin (GAP43) (Fig. 1). The other protein clusters (*n* = 18 and *n* = 3) showed generally lower co-variation between proteins (median ρ 0.54, IQR 0.23, and median ρ 0.34, IQR 0.14, respectively). Neurofilament medium (NEFM), being in the small cluster of three proteins, did not exhibit strong correlations with the neuronal proteins in the first cluster (median ρ 0.20, IQR 0.18). Furthermore, chitinase 1 (CHIT1), included in the cluster together with NEFM, displayed a unique correlation profile (median *ρ* -0.01, range − 0.08 − 0.07, IQR 0.04).

### CSF proteins are associated with ECAS total score

We applied elastic net as a variable selection method to identify proteins with a relevant association to ECAS total score. In our final elastic net model, the optimal hyperparameters were found to be α = 0.54 and λ = 3.63. This indicated that the final model used a nearly balanced mix of LASSO and ridge regression penalties, reflecting both the need for variable selection and stability in the presence of potential multicollinearity. The model identified 7 proteins as the most predictive of ECAS total score: NEFM, neuronal pentraxin 2 (NPTX2), GAP43, insulin like growth factor binding protein 4 (IGFBP4), insulin like growth factor binding protein 7 (IGFBP7), osteopontin (SPP1) and cadherin 8 (CDH8) (Table 2). The model produced an RMSE of 13.76 on the training set and 12.03 on the test set. The slightly lower RMSE on the test set suggests that the model not only fit the training data well but also generalized effectively to unseen data, with no indications of overfitting.

**Table 2:** Candidate protein and protein pairs. Results from the elastic net and linear regression analyses

	Linear regression		Selected in elastic net of single proteins	Selected in elastic net of protein pairs
	β coefficient	CV *R*^2^		
Single protein				
IGFBP7	4.05 (0.11–7.98)	0.27	Yes	No
NPTX2	2.83 (-1.09–6.76)	0.29	Yes	Yes
CDH8	2.57 (-1.37–6.50)	0.25	Yes	Yes
PTPRN2	2.26 (-1.82–6.34)	0.24	No	Yes
IGF2	2.07 (-2.04–6.19)	0.13	No	Yes
CHL1	1.62 (-2.52–5.76)	0.26	No	Yes
CADM2	1.07 (-3.03–5.16)	0.34	No	Yes
BASP1	0.67 (-3.50–4.84)	0.18	No	Yes
IGFBP4	-0.06 (-4.37–4.24)	0.21	Yes	Yes
GAP43	-1.25 (-5.41–2.91)	0.23	Yes	Yes
NEFM	-2.58 (-6.92–1.77)	0.22	Yes	No
SPP1	-3.45 (-7.34–0.45)	0.47	Yes	Yes
Protein pair				
PTPRN2/GAP43	7.48 (3.66–11.29)	0.34		
CDH8/GAP43	6.77 (2.68–10.85)	0.32		
CHL1/GAP43	6.49 (2.38–10.61)	0.30		
NPTX2/SPP1	5.24 (1.49–8.99)	0.26		
PTPRN2/BASP1	4.94 (0.66–9.22)	0.19		
CADM2/GAP43	3.5 (-0.63–7.63)	0.22		
NPTX2/IGFBP4	3.05 (-1.27–7.37)	0.28		
IGF2/IGFBP4	2.92 (-1.24–7.07)	0.27		

*CI* confidence interval, CV *R*^2^= 10-fold cross-validated *R*^2^

### Protein ratios are superior to single proteins for predicting ECAS score

**Fig. 2:**
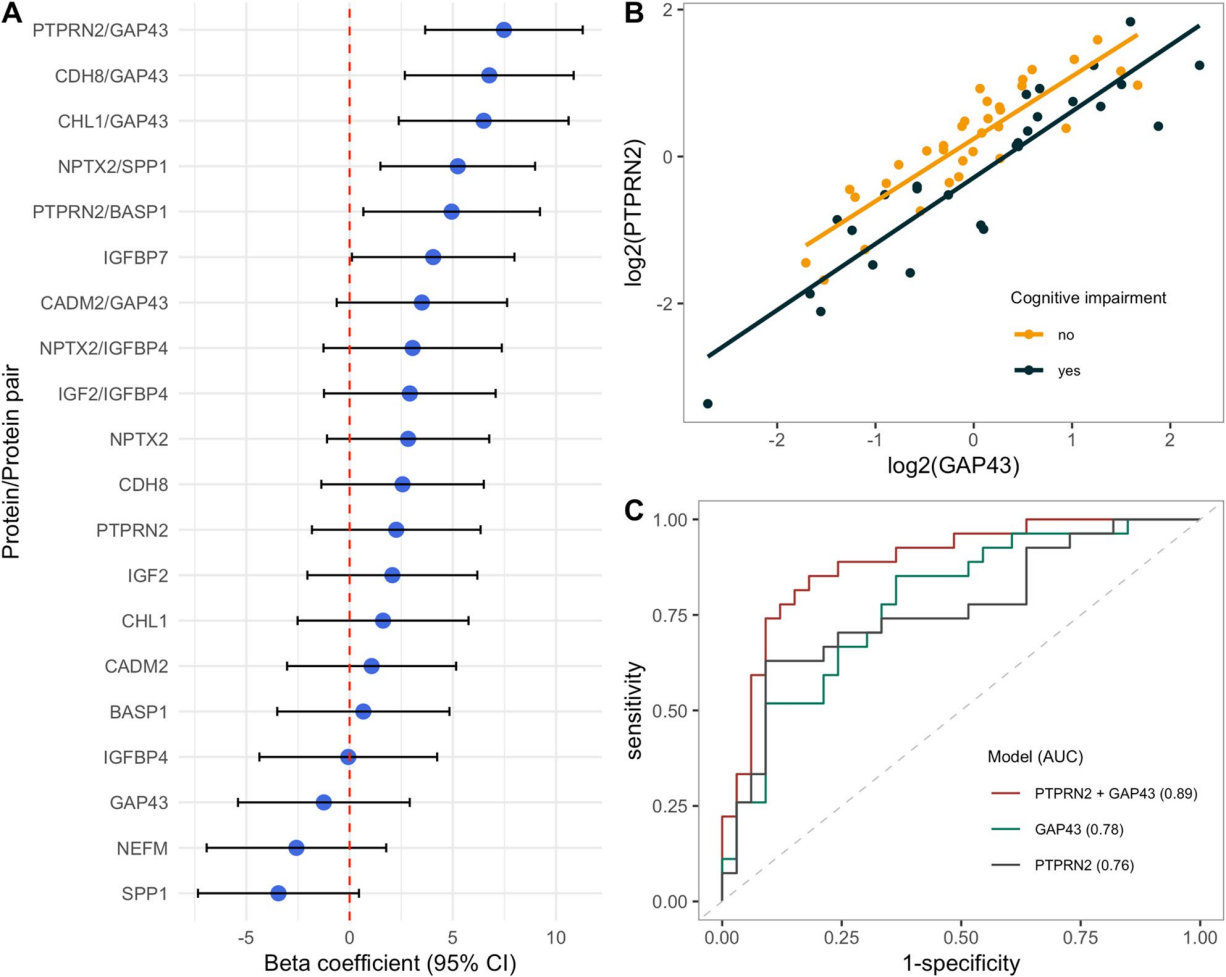
**A** Forest plot showing associations to ECAS total score for protein/protein pair candidates. Each blue point represent the linear regression model β coefficient and the line the corresponding confidence interval. All models were adjusted for age at sampling, sex, site of onset and ALSFRS-R. The red dashed line correspond to a β coefficient of 0. **B** Relationship between PTPRN2 and GAP43 coloured by cognitive status. **C** ROC curve showing the predictive performance for ALS with vs. without cognitive impairment. Illustrating three different analyses: GAP43 + PTPRN2 and GAP43 and PTPRN2 alone, all adjusted for age at sampling, sex, site of onset and ALSFRS-R total score

As protein pairs have been shown to provide stronger associations with cognitive function compared to single proteins in other neurodegenerative disorders, we also assessed protein ratios for their ability to detect cognitive impairment in ALS. All 47 proteins were combined into pairs (*n* = 2162). Again, we used elastic net to find the pairs most predictive of ECAS total score. In the elastic net model of ratios, the optimal hyperparameters were found to be α = 0.37 and λ = 10.38. Here, 8 protein pairs were identified as the most predictive of ECAS total score (Table 2; Fig. 2A). The model produced an RMSE of 12.93 on the training set and 12.11 on the test set.

Five of the proteins selected in the ratio elastic net model were also selected in the single protein model (NPTX2, GAP43, IGFBP4, SPP1 and CDH8). Interestingly, five additional proteins were found relevant as part of a pair, namely insulin like growth factor 2 (IGF2), cell adhesion molecule L1 like (CHL1), protein tyrosine phosphatase receptor type N2 (PTPRN2), cell adhesion molecule 2 (CADM2) and brain abundant membrane attached signal protein 1 (BASP1). As previously shown, neither of these had a strong association with ECAS total score alone.

To further evaluate the association to ECAS total score and compare the performance of single proteins and protein pairs, linear regression models were created for each candidate as the predictor (Table 2; Fig. 2A). We assessed the predictive performance of each model using 10-fold cross-validated *R*² (CV *R*²). Comparing the regression models revealed that higher CV *R*² values were generally observed in protein pair models (median 0.27) in contrast with those of the single protein models (median 0.24) (Supplementary Fig. 3). In particular, protein pair ratios involving GAP43 exhibited larger β coefficient magnitudes, the majority of the confidence intervals excluded zero, and higher cross-validated *R*² values relative to single protein metrics (Table 2). The PTPRN2/GAP43 ratio showed the strongest association with an estimated β coefficient of 7.48 (95% CI: 3.66–11.29) with a 10-fold cross-validated *R*² of 0.34. Other prominent protein pairs included CDH8/GAP43, CHL1/GAP43 and NPTX2/SPP1. The PTPRN2/GAP43 β coefficient substantially exceeded that of PTPRN2 and GAP43 alone (2.26, 95% CI: -1.82–6.34 and − 1.25 (95% CI: -5.41–2.91, respectively), suggesting that the ratio of these highly correlating proteins is more informative than the levels alone (Fig. 2B). This additive effect of PTPRN2 in combination with GAP43 was further explored using ROC analysis (Fig. 2C). The performance was considerably better for PTPRN2/GAP43, with an area under the curve of 0.89 (95% CI: 0.80–0.97), compared to PTPRN2 and GAP43 alone (AUC 0.76 95% CI: 0.64–0.89 and 0.78 95% CI: 0.67–0.90, respectively). These results suggest that protein ratios potentially offer a more robust prediction of ECAS total score compared to single protein measures. The consistency of key marker selection in both elastic net and linear regression analyses further reinforces the potential of these ratios as biomarkers for cognitive impairment in ALS.

We next evaluated the association of the most promising protein ratios with ECAS sub scores. The strongest associations with executive function, verbal fluency, and memory for the PTPRN2/GAP43 ratio (Supplementary Fig. 4). Similar results were found for the other candidate pairs suggesting that these ratios are more likely markers of a general cognitive dysfunction and not specific to ALS frontotemporal involvement.

### The trajectories of CSF PTPRN2/GAP43 are different in males and females with cognitive impairment

**Fig. 3:**
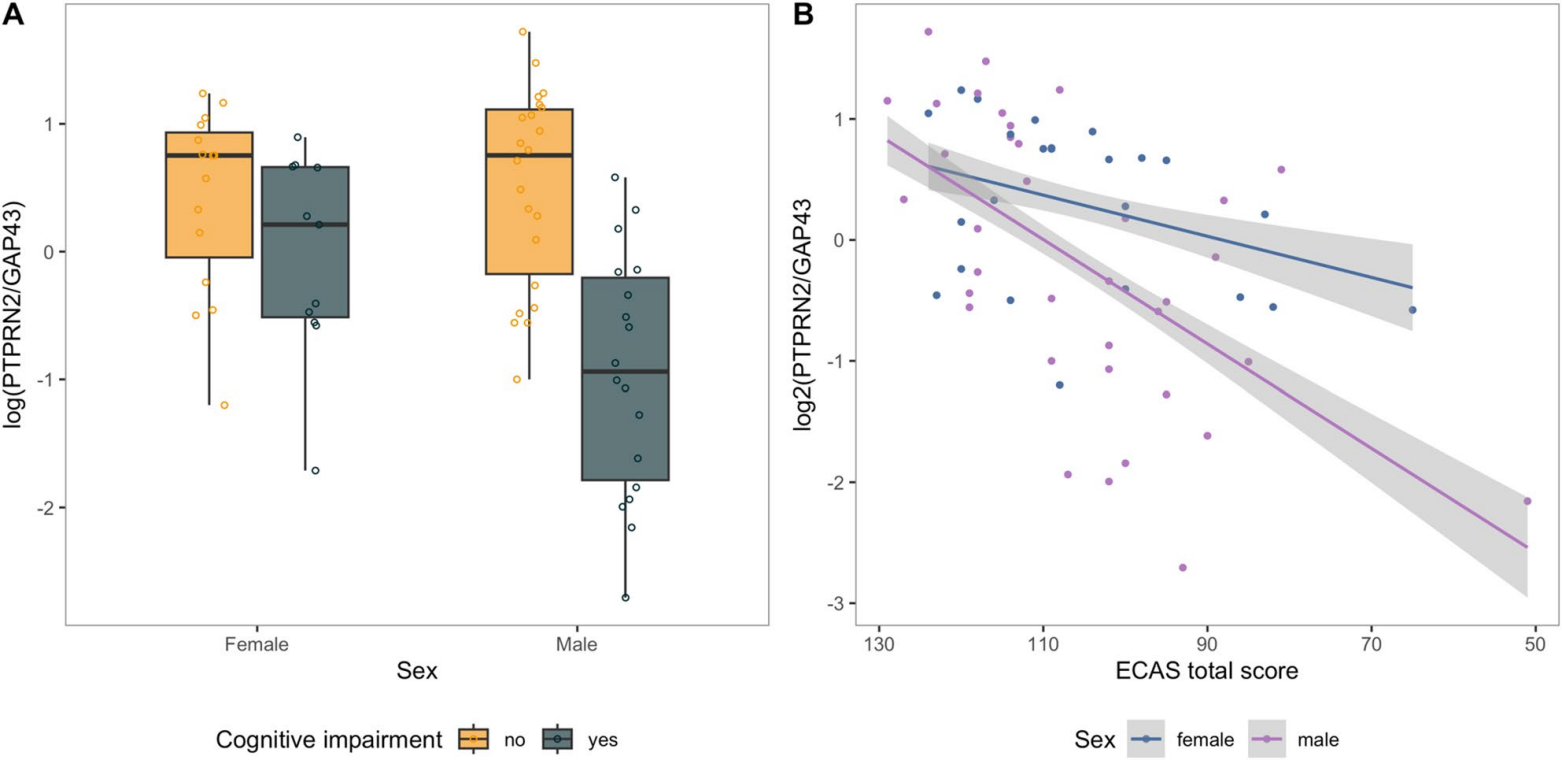
The association between PTPRN2/GAP43 and sex.** A** Boxplot of PTPRN2/GAP43 for female and men with and without cognitive impairment. **B** Scatterplot of PTPRN2/GAP43 over ECAS total score coloured by sex. Grey, shadowed areas represent the standard error. The model included ECAS total score and sex as covariates including an interaction term between these two variables, and was adjusted for age at sampling, site of onset, and ALSFRS-R

The PTPRN2/GAP43 ratio was not associated with ALSFRS-R score, nor were there any statistically significant differences in the PTPRN2/GAP43 CSF ratio between bulbar versus spinal onset or *C9orf72* mutation carriers versus non-carriers (Supplementary Fig. 5A, Supplementary Fig. 5B). In addition, we did not find any overall differences in PTPRN2/GAP43 ratio between the sexes (*p* = 0.16). However, among the patients with cognitive impairment, lower ratios were found in males compared to females even though the distribution of cognitive impairment and age was similar between sexes (*p* = 0.002) (Fig. 3A, Supplementary Fig. 6). Indeed, when exploring linear regression with PTPRN2/GAP43 as the outcome and including an interaction term between ECAS total score and sex, we found that the slope of PTPRN2/GAP43 ratio over ECAS total score was steeper for males than females (0.04 and 0.01 respectively, on a log2 scale) (Fig. 3B).

## Discussion

In this study we analysed a panel of CSF proteins and investigated their association to cognitive function in patients with ALS. No single protein strongly predicted cognitive impairment but combining them into ratios markedly improved predictive power. This resonates with findings in AD, where ratios like Aβ42/40 or Aβ42/tau better reflect disease processes than individual proteins [25–27]. Such ratios might capture interactions between pathways, for example synaptic repair (GAP43) and metabolic alterations (PTPRN2). A possible explanation for the enhanced performance of protein pairs in relation to single proteins is that one protein in the ratio act as a reference, thereby accounting for non-disease related individual variation. Prior studies have suggested that such variability can be influenced by factors like sex, age, and ventricular volume [8, 17]. The separation between patients with and without cognitive impairment in Fig. 2B suggests that patients with impairment tend to have lower PTPRN2 levels relative to GAP43, supporting the ratio as an informative readout of a relative shift between two correlated markers rather than an effect of PTPRN2 alone.

Variable selection methods like elastic net can be used to highlight proteins with important contributions to a multivariable model, but their performance may not always translate into regression models. In our case, this means that while these proteins are informative in a multivariate setting, they may not independently explain cognitive variance when clinical variables are accounted for. This could explain why several of the identified single proteins were selected in the elastic net, even though they did not demonstrate strong predictive performance in the subsequent adjusted linear models.

In this study, the most promising candidate biomarker associated with cognitive impairment was the PTPRN2/GAP43 ratio, the same protein pair previously found to be among the best classifiers of cognitive decline in patients with dementia [3, 6]. While levels of GAP43 have been associated with AD in previous studies (including [15,16,19,28–30]), the function and role of PTPRN2 in neurodegenerative disease pathology is poorly understood. In a clinical setting, complex multivariable models with many covariates and interaction terms can be difficult to apply or interpret, limiting their practical use. In contrast, simpler measures, like ratios of two proteins, are more feasible for clinical translation, as they balance complexity with interpretability and robustness. Our findings that protein ratios outperform single markers are in line with recent publications and these studies together suggest that the use of protein pairs might be a suitable middle way for practical biomarker implementation [4, 6]. Unlike established AD ratios (e.g. Aβ42/40), most protein ratios should not be assumed to reflect a single pathological process. While ratios can be motivated by functional pairing or by reference effects, we interpret the main advantage here as improved robustness, capturing relative shifts between correlated proteins and reducing shared variance and inter-individual variability, while any mechanistic interpretation remains speculative. Due to the cross-sectional design, we cannot determine whether the ratios are predictive of subsequent cognitive decline. Longitudinal studies with repeated cognitive assessments and serial biomarker sampling are required to evaluate sensitivity to emerging cognitive change, estimate predictive performance, and determine whether biomarker trajectories precede clinical cognitive impairment and thus support early diagnosis or risk stratification.

Our cluster analysis revealed a strong co-variation between many of the CSF proteins relevant for synaptic health and function. The tight intercorrelations within the largest cluster suggest that these proteins may be co-regulated or share common biological pathways involved in neurodegeneration. Neurofilament medium, a marker of axonal integrity closely related to the well-established neurofilament light chain [10], did not strongly correlate with these cognitive markers and may be involved in only partially reflect overlapping pathological mechanisms. The clustering pattern of NEFM instead suggests stronger association with neuroinflammation or immune response rather than synaptic function. Together with the finding that protein pairs including NEFM were not selected as key features in the elastic net regression, indicate that cognitive dysfunction in ALS might be more closely driven by synaptic and cortical processes than by general axonal damage.

The observed sex differences in CSF PTPRN2/GAP43 levels in relation to cognitive impairment are particularly noteworthy. Specifically, our regression analysis revealed a significant interaction between sex and ECAS total score, with a markedly steeper decline in the PTPRN2/GAP43 ratio among males. Although this ratio does not independently predict ECAS scores when modelled in the reverse direction, the interaction suggests that cognitive status is more strongly associated with biomarker levels in males. This finding not only reinforces the group differences seen in our stratified analyses but also points to a potentially meaningful biological divergence. It raises the possibility of sex-specific dynamics in biomarker expression or regulation in the context of ALS-related cognitive impairment. Further investigation is needed to determine whether these differences reflect distinct underlying mechanisms or differential susceptibility to cognitive decline between sexes.

A strength of this study lies in its rigorous application of statistical methods and cross-validation to reduce overfitting and assess internal model stability. However, one limitation is the small sample size, especially for sex-stratified analyses, which may limit the generalisability of the results. Therefore, external replication in an independent ALS cohort is necessary before these biomarkers can be considered for clinical translation. Another limitation is the targeted nature of the proteomic analysis. Another limitation is the targeted nature of the proteomic analysis. The protein panel was curated primarily based on prior evidence from AD and was chosen to test whether previously identified CSF markers generalise to ALS. This introduces a potential selection bias toward proteins reflecting shared neurodegenerative and neuroinflammatory pathways and may underrepresent proteins that are more specific to ALS biology, including neurotransmitter-specific mechanisms (e.g. glutamatergic excitotoxicity). Consequently, relevant ALS-specific markers may have been missed. Furthermore, while ECAS is a validated screening tool for cognitive impairment in ALS, our cut-offs were not adjusted for potential confounders such as age and education, which may influence cognitive performance and the classification of impairment [31]. While the strong performance of CSF PTPRN2/GAP43 suggests biological relevance, future studies should aim for larger cohorts and more comprehensive cognitive assessments to confirm these findings.

## Conclusions

Altogether, our data support the hypothesis that protein ratios, particularly PTPRN2/GAP43, may capture aspects of cognitive impairment in ALS that go undetected by single markers. They also highlight the importance of considering sex-specific interactions when interpreting biomarker data. If validated in larger cohorts, these findings could aid the development of diagnostic methods in ALS that are sensitive to cognitive impairment and argue for prioritising combinatorial or ratio-based measures over single protein readouts in future biomarker panels. Such panels could also have broader implications, as similar synaptic processes are implicated in other neurodegenerative diseases, suggesting shared targets for therapeutic intervention.

## Supplementary Information

Supplementary Material 1.

## Data Availability

The dataset used and analysed during the current study are available from the corresponding author upon reasonable request.

## Abbreviations

AD: Alzheimer disease
ALS: Amyotrophic lateral sclerosis
ALSFRS-R: ALS functional rating scale–revised
AUC: Area under the curve
BASP1: Brain abundant membrane attached signal protein 1
C9orf72: C9 open reading frame 72
CADM2: Cell adhesion molecule 2
CDH8: Cadherin 8
CHIT1: Chitinase 1
CHL1: Cell adhesion molecule l1 like
CI: Confidence interval
CSF: Cerebraospinal fluid
CV: Cross validation
ECAS: Edinburgh cognitive and behavioural ALS screen
FTD: Frontotemporal dementia
GAP43: Neuromodulin
IGF2: Insulin like growth factor 2
IGFBP4: Insulin like growth factor binding protein 4
IGFBP7: Insulin like growth factor binding protein 7
IQR: Inter quartile range
LASSO: Least absolute shrinkage and selection operator
NEFM: Neurofilament medium
NPTX2: Neuronal pentraxin 2
NRGN: Neurogranin
PTPRN2: Protein tyrosine phosphatase receptor type n2
RMSE: Root mean squared error
ROC: Receiver operating characteristics
SNCB: Beta-synuclein
SPP1: Osteopontin
