# Supplementary material for: Ratios of CSF proteins reflect cognitive function in ALS
Source: Alzheimers Res Ther. 2026 Jan 31;18:46. doi: 10.1186/s13195-026-01976-y (PMC12931033; doi:10.1186/s13195-026-01976-y)

SUPPLEMENTARY

Ratios of CSF Proteins Reflect Cognitive Function in ALS

Linn Öijerstedt[1,2], Sára Mravinacová[3], Jennie Olofsson[3], Louisa Azizi[1], Sofia Bergström[3], Solmaz Yazdani[1], Nina De Vita[3], Inci S. Aksoylu[3], Juliette Foucher[1,2], Alexander Juto[1,2], Ulf Kläppe[1,2], Peter Nilsson[3], Anna Månberg[3] and Caroline Ingre[1,2]

1. Department of Clinical Neuroscience, Karolinska Institutet, Stockholm, Sweden.
2. Department of Neurology, Karolinska University Hospital, Stockholm, Sweden.
3. Department of Protein Science, KTH Royal Institute of Technology, SciLifeLab, Stockholm, Sweden.

Supplementary Table 1. Proteins included.

Antibody ID	Gene	Gene description	Uniprot ID
HPA019829	AMPH	amphiphysin	P49418
M067-3	APOE4	Apolipoprotein E 4	P02649
HPA014784	AQP4	aquaporin 4	P55087
HPA017303	ARPP21	cAMP regulated phosphoprotein 21	Q9UBL0
HPA050333	BASP1	brain abundant membrane attached signal protein 1	P80723
HPA046356	C4B;C4A	complement C4B (Chido blood group);complement C4A (Rodgers blood group)	P0C0L5;P0C0L4
HPA010024	CADM2	cell adhesion molecule 2	Q8N3J6
HPA069515	CCK	cholecystokinin	P06307
HPA002035	CD14	CD14 molecule	P08571
HPA014908	CDH8	cadherin 8	P55286
HPA010575	CHIT1	chitinase 1	Q13231
HPA003345	CHL1	cell adhesion molecule L1 like	O00533
HPA012749	CLSTN1	calsyntenin 1	O94985
HPA002988	CTSS	cathepsin S	P25774
HPA006308	DDAH1	dimethylarginine dimethylaminohydrolase 1	O94760
HPA013603	GAP43	growth associated protein 43	P17677
AF2420	GRN	Progranulin	P28799
HPA072414	IGF1	insulin like growth factor 1	P05019
HPA007556	IGF2	insulin like growth factor 2	P01344
HPA046972	IGFBP1	insulin like growth factor binding protein 1	P08833
HPA045140	IGFBP2	insulin like growth factor binding protein 2	P18065
HPA066240	IGFBP4	insulin like growth factor binding protein 4	P22692
HPA059827	IGFBP5	insulin like growth factor binding protein 5	P24593
HPA075088	IGFBP6	insulin like growth factor binding protein 6	P24592
HPA002196	IGFBP7	insulin like growth factor binding protein 7	Q16270
HPA001615	KNG1	kininogen 1	P01042

HPA049222	MBP	myelin basic protein	P02686
HPA021873	MOG	myelin oligodendrocyte glycoprotein	Q16653
HPA058000	NCAN	neurocan	O14594
HPA022845	NEFM	neurofilament medium chain	P07197
HPA077062	NPTX1	neuronal pentraxin 1	Q15818
HPA049799	NPTX2	neuronal pentraxin 2	P47972
HPA001079	NPTXR	neuronal pentraxin receptor	O95502
HPA061433	NRCAM	neuronal cell adhesion molecule	Q92823
HPA038171	NRGN	neurogranin	Q92686
HPA008206	OMG	oligodendrocyte myelin glycoprotein	P23515
HPA042260	PAM	peptidylglycine alpha-amidating monooxygenase	P19021
HPA053342	PDYN	prodynorphin	P01213
HPA063904	PEBP1	phosphatidylethanolamine binding protein 1	P30086
HPA007255	PTPRN2	protein tyrosine phosphatase receptor type N2	Q92932
HPA002475	RPH3A	rabphilin 3A	Q9Y2J0
HPA035876	SNCB	synuclein beta	Q16143
HPA027541	SPP1	secreted phosphoprotein 1	P10451
HPA070770	TARDBP	TAR DNA binding protein	Q13148
HPA010739	TMEM132D	transmembrane protein 132D	Q14C87
HPA005993	UCHL1	ubiquitin C-terminal hydrolase L1	P09936
HPA055177	VGF	VGF nerve growth factor inducible	O15240

Supplementary Figure 1. **Schematic illustration of the elastic net.**

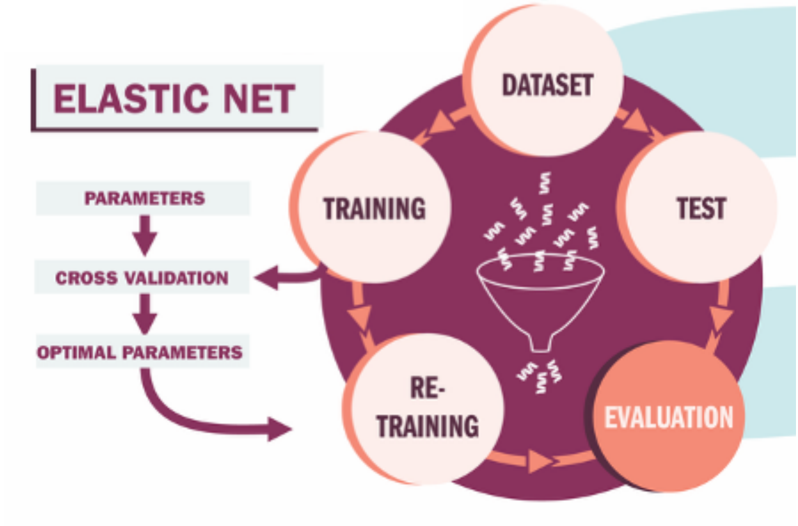

Supplementary Figure 2. **CV plot for choosing optimal lambda in elastic net of A) single proteins and B) protein pairs.**

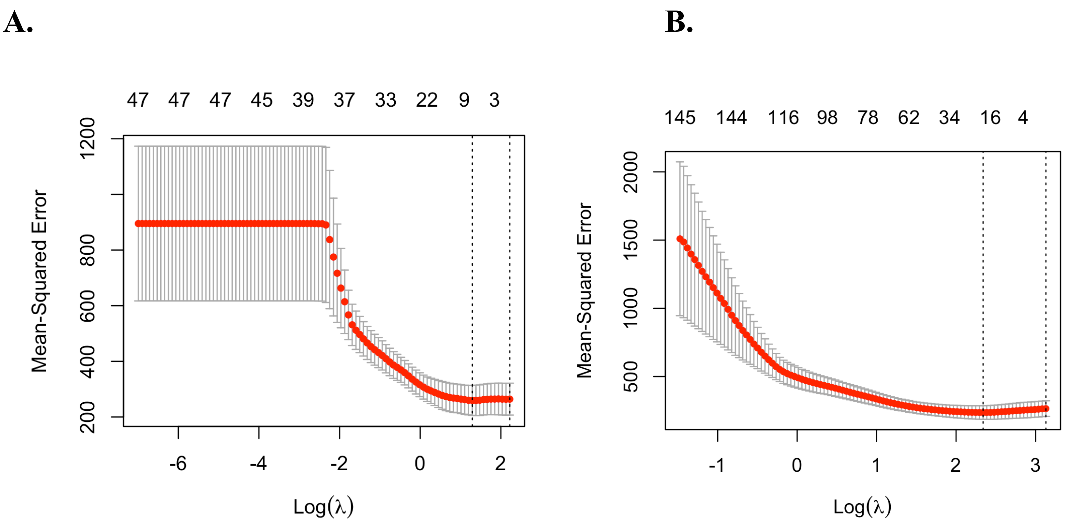

Supplementary Figure 3. **Density plots comparing beta coefficients for ratios vs single proteins for ECAS total score and subscores.**

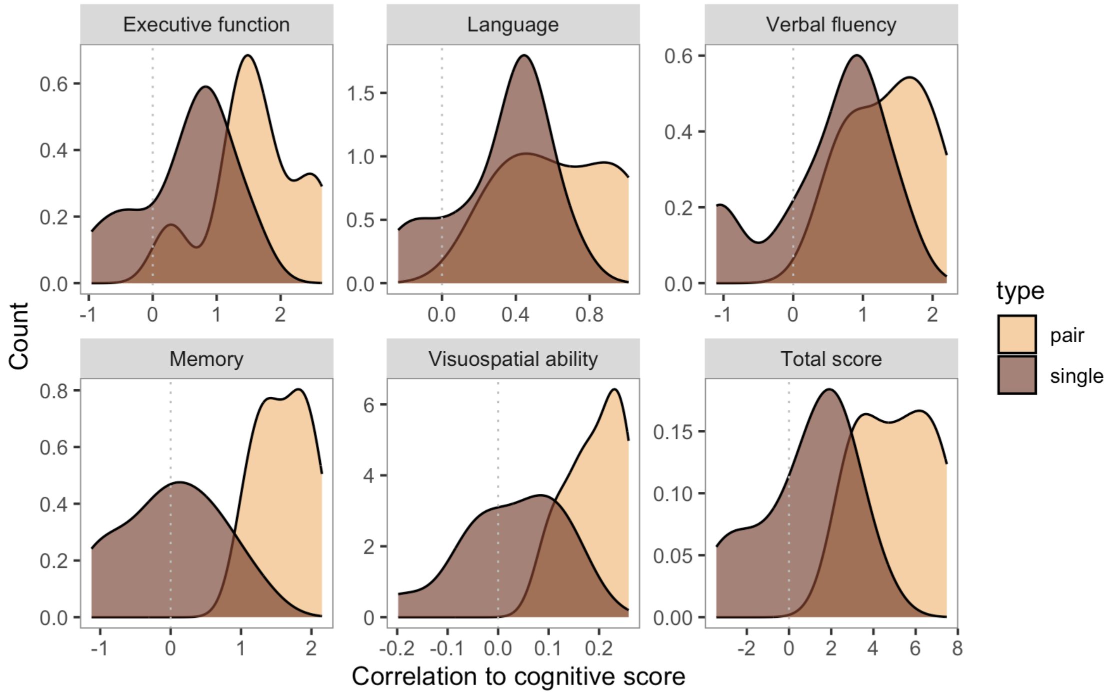

Supplementary Figure 4. **Relationships between all candidate ratios and ECAS subscores.**

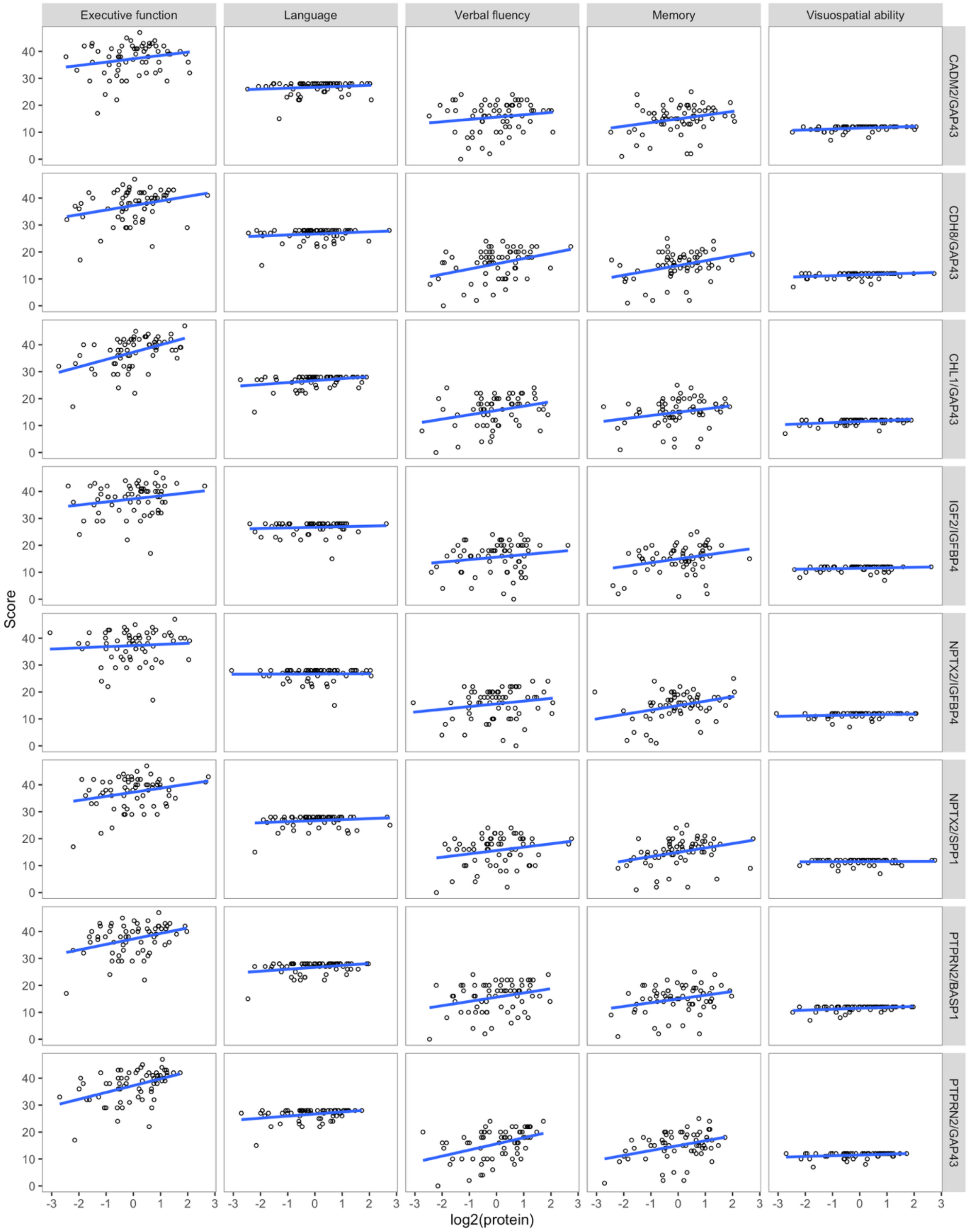

Supplementary Figure 5. **The association between PTPRN2/GAP43 and A) site of onset (other = respiratory) and B) genetic status (MC=carrier of the *C9orf72* repeat expansion, NC=non-carrier).**

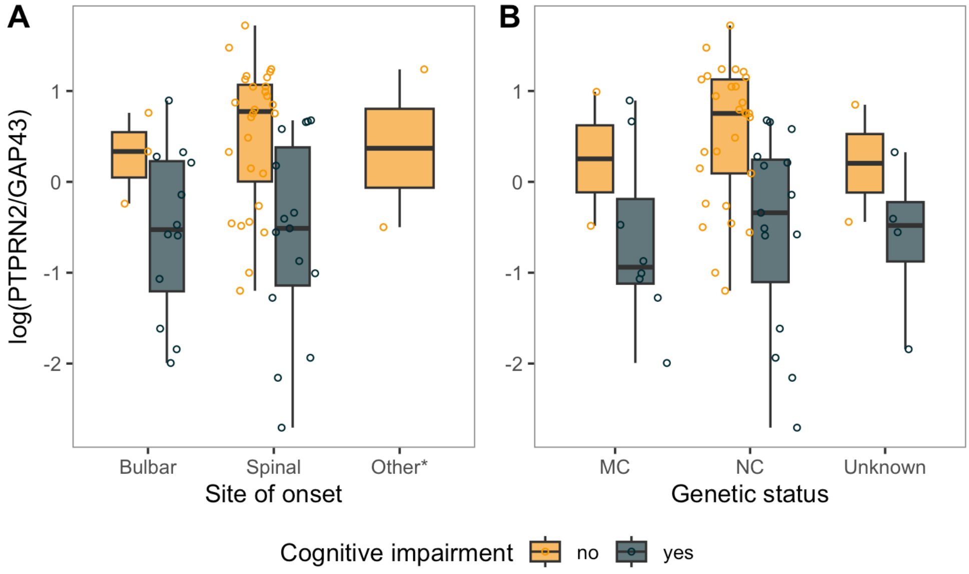

Supplementary Figure 6. **Relationship between PTPRN2 and GAP43 for females and males, coloured by cognitive status.**

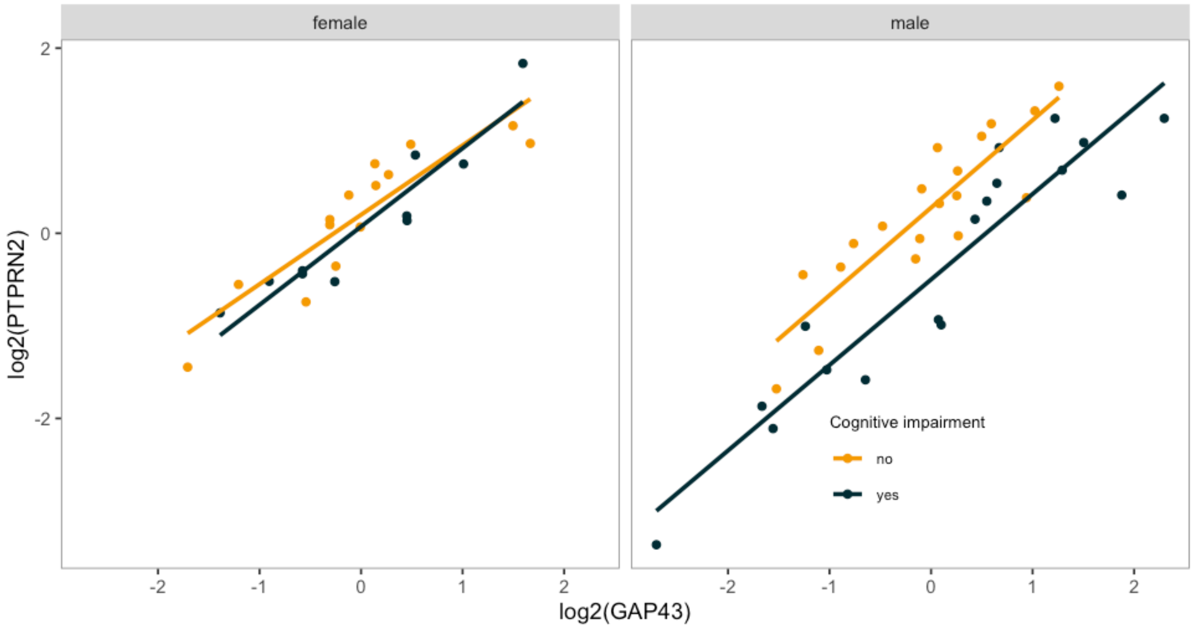

Supplement: Supplementary file 1 — Supplementary Material 1. [file 13195_2026_1976_MOESM1_ESM.pdf]
